# BMSC seeding in different scaffold incorporation with hyperbaric oxygen treats seawater-immersed bony defect

**DOI:** 10.1186/s13018-021-02368-8

**Published:** 2021-04-13

**Authors:** Gan Zhang, Xiaosong Chen, Xunsheng Cheng, Wuxiu Ma, Congcong Chen

**Affiliations:** Department of Orthopaedics, The 901th Hospital of the Joint Logistics Support Force of the Chinese People’s Liberation Army, Hefei, 230031 China

**Keywords:** BMSCs, HBO, Seawater-immersed bone defect, n-HA, β-TCP, PLGA

## Abstract

**Introduction:**

The experiment was undertaken to estimate the effect of BMSC seeding in different scaffold incorporation with HBO on the repair of a seawater-immersed bone defect. And future compared n-HA/PLGA with β-TCP/PLGA as a scaffold in treatment effect of the seawater-immersed bone defect.

**Methods:**

Sixty New Zealand White rabbits with standard seawater defect in radius were randomly divided into group A (implant with nothing), group B (implanted with autogenous bone), group C (implanted with n-HA/PLGA/BMSCs), and group D (implanted with β-TCP/PLGA/BMSCs). After the implant, each rabbit receives HBO treatment at 2.4 ATA 100% oxygen for 120 min/day for 2 weeks. Radiograph, histological, and biomechanical examinations were used to analyze osteogenesis.

**Result:**

X-ray analysis shows that n-HA/PLGA/BMSCs and β-TCP/PLGA/BMSCs could accelerate the new bone formation, and the new bone formation in group C was larger than that in group D or group A and close to group B (*P* < 0.05). After 12 weeks, in group A, the defect without scaffold shows a loose connect tissue filled in the areas. The medullary canal in group B was recanalized. Defects in groups C and D show a larger number of woven bone formation. The new woven bone formation in defect areas in group C was larger than that in group D. The mechanical examination revealed ultimate strength at 12 weeks was group D > group C > group B > group A (*P* < 0.05).

**Conclusion:**

Scaffolds of n-HA/PLGA and β-TCP/PLGA incorporation with HBO and BMSCs were effective to treat seawater-immersed bone defect, and n-HA/PLGA was more excellent than β-TCP/PLGA.

**Supplementary Information:**

The online version contains supplementary material available at 10.1186/s13018-021-02368-8.

## Introduction

Healing bone defects was a difficult problem in orthopedics. When a bone defect was immersed in seawater, the repairing becomes more difficult. Autologous graft and allograft were possible methods. An autologous bone graft is regarded as the gold standard in treating bone defects. However, the donor site bone source is limited, obtaining the autogenous bone was an invasive operation, which can lead to high donor site morbidity [1, 2]. Allograft has the potential of transmission of diseases and leading to immune response. Bone tissue engineering was a promising method that can overcome these problems mentioned above.

Poly lactic-co-glycolic acid (PLGA) was widely used in bone tissue engineering due to its nontoxic and biodegrade as a scaffold. PLGA has been approved by the Food and Drug Administration for human clinical applications [3]. But it has poor mechanical strength and cell affinity which limits its use in bone tissue engineering [4–6]. In addition, acidic degradation can lead to inflammatory reactions [7, 8]. McBane et al. reported that PLGA films were implanted subcutaneously in a rodent model which caused acute inflammatory response [9]. PLGA scaffold has successfully repaired the bone defect in SD [10]. However, PLGA is not an excellent scaffold in bone tissue engineering owing to its hydrophobicity and lack of bioactive properties and its degradation products lead to inflammation. Its properties can be improved by combining with other materials. In our study, there are two materials to be tried: (1) n-HA and (2) β-TCP.

HBO therapy is exposing patients to 100% oxygen under elevated pressure. Jan et al. [11] evaluated autogenous bone graft and autogenous bone graft with HBO by micro-CT analysis and histomorphometric analysis and found that new bone formation was more in autogenous bone graft with HBO, suggesting that HBO can enable the bony healing of the critical-sized bone defect. HBO therapy is thought to increase healing by increasing the amount of oxygen dissolved in the blood. In Grassmann et al. [12], HBO therapy enhances bone repairing, which may attribute to an increase in angiogenesis.

## Material and methods

### Experimental main material and reagents

BMSCs, MSC growth medium, and trypsin were purchased from Cyagen Biosciences Inc. (Guangdong, China). The n-HA/PLGA and β-TCP/PLGA scaffolds (4 mm × 15 mm, cylinder) were provided by The Shandong Province Key Laboratory of Medical Polymer Materials (Jinan, China).

### Cell culture

BMSCs were obtained from Cyagen at passage 2. The BMSCs were thawed and cultured in a growth medium from Cyagen in a humidified atmosphere containing 5% CO_2_ at 37 °C. The growth medium contained 440 ml MSC basal medium, 50 ml 10% FBS, and penicillin/streptomycin mixture. The growth medium was changed every 2–3 days. The cells were passaged 3 times at approximately 80% confluence. Passage 5 BMSCs were digested and collected for determination and culture with the scaffold.

### Cell seeding into the scaffold

The PLGA/n-HA and gelatin/n-HA were treated with 75% alcohol and then washed 3 times using phosphate-buffered saline (PBS). BMSCs at 5 passage were suspended in a growth medium at 2 × 10^7^ cells/ml. Two hundred microliters of cell suspension was dropped on the top of the scaffold. The scaffold with cells was cultured in cutie for 2 h and then set in a 24-well plate with the growth medium. The medium was changed every 2–3 days.

### Animal

Sixty New Zealand White rabbits (weighing 2.0–3.0 kg) were obtained from the animal experiment center of Anhui Medical University (Hefei, China). Before the experiments, approval was obtained from the Ethical Committee for animal experiments of Anhui Medical University. The rabbits were anesthetized by ear vein of 3% pentobarbital sodium (30 mg/kg). Disinfection was done with iodine and 75% alcohol. A 3–5-cm incision was created in the middle of the radius. The tissue overlying the radius was dissected. A 15-mm bony defect was made in the middle of the radius. The forelimbs with 15-mm bone defect were immersed in seawater for 3 h. The rabbits with seawater-immersed bone defect were divided into 4 groups (group A, group B, group C, and group D), with 15 rabbits in each group (*n* = 15). Group C was implanted with n-HA/PLGA/BMSCs, group D was implanted with β-TCP/PLGA/BMSCs, group B was implanted with autograft obtained from the iliac crest, and group A was implanted with anything. After implantation, the rabbits were treated with 2.4 ATA 100% HBO for 90 min/day for 2 weeks. The rabbits were injected intramuscularly with penicillin every day for 3 days.

### Radiographic examination

Radiographs of rabbit radius were examined at 4, 8, and 12 weeks after surgery under anesthesia. Radiological evaluation was done using the Lane and Sandhu scoring system [13]. The radiographs were evaluated by orthopedists. And the evaluation was under a double-blinded study.

### Histopathologic examination

After 4, 8, and 12 weeks, the rabbits were killed by euthanasia. Samples harvested from radius defect sites were fixed in 10% neutral buffered formalin for 48 h. The samples were decalcified with 10% EDTA solution for 30 days, dehydrated in graded ethanol, and embedded in paraffin. Five-micrometer sections were cut and stained with hematoxylin-eosin.

### Immunohistochemistry stain of osteocalcin

Immunohistochemical examination of OCN was performed by the slide deparaffinized in xylene I–II respectively for 15 min and dehydrated in grade alcohol from 90 to 70% for 3 min. After that, blocking was done with 0.5% H_2_O_2_ in methanol for 30 min and washed with water for 5 min. Pretreatment of the slide was performed with citrate buffer in microwave cook I and cook II for each 5 min followed by blocking background target to block non-specific antigens and then incubation for 15 min. Then, it was given primary antibody to OCN and incubated for 1 h. The slide was given a universal ink secondary antibody to bind to the primary antibody for 15 min. Counterstaining was performed with hematoxylin for 1–2 min.

### Bone mechanical strength test

Twelve weeks after surgery, the mechanical strength of the radius was tested by the three-point bending test. The test was performed using a universal tensile testing machine (Instron, London, UK). The ultimate force in the bending test is until the specimen was broken. The bones were placed horizontally on two rounded supporting bars located at a distance of 30 mm, and the bending load was applied at the midpoint of the defect at the loading speed of 10 mm/min until the specimen fracture. The biomechanical properties of the specimens were determined by ultimate loading (N). The data were recorded as the mean plus standard error of the mean.

### Statistical analysis

All data are presented as mean ± SD. Comparisons between groups were done by one-way analysis of variance (ANOVA). The SPSS 19.0 was used for statistical analysis. The differences were considered as statistical significance at the level of *P* < 0.05.

## Result

### Radiological examination

**Fig. 1:**
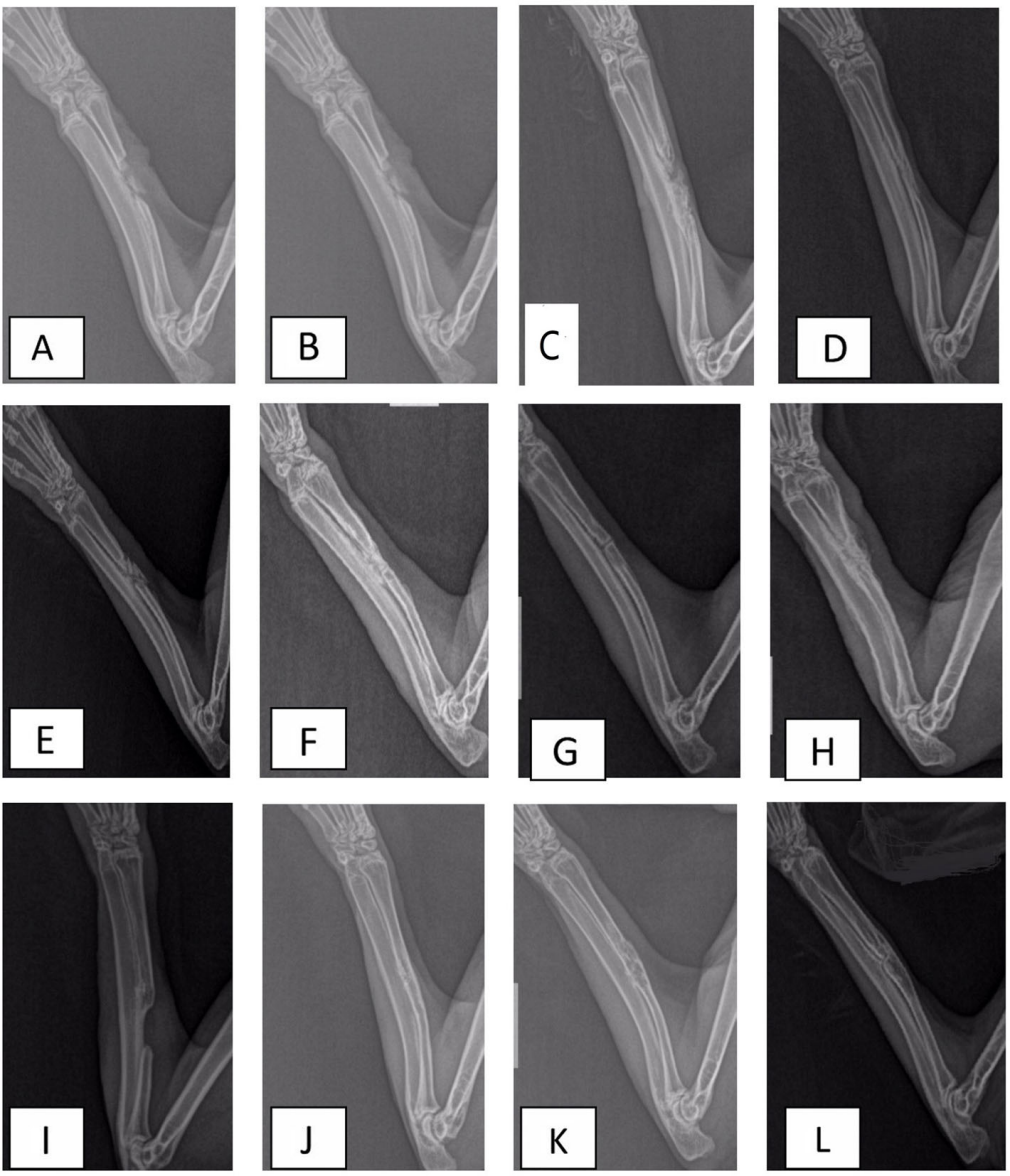
Radiographs in A group after operation 4 weeks (**a**), 8 weeks (**e**), and 12 weeks (**i**); B group after operation 4 weeks (**b**), 8 weeks (**f**), and 12 weeks (**j**); C group after operation 4 weeks (**c**), 8 weeks (**g**), and 12 weeks (**k**); and D group after operation 4 weeks (**d**), 8 weeks (**h**), and 12 weeks (**l**)

X-ray examinations were done to evaluate the development of bone regeneration in the defect, which are displayed in Fig. 1. At 4 weeks after surgery, litter callus formatted in group A. The shadow of autogenous bone in group B was visible and callus formatted in the ends of the bone defect. The shadow of the area of bone defect in groups C and D was filled with bony callus, which was observed as the cloudy shadow. The area of cloudy shadow in group C was larger than that in group D. After 8 weeks, the bony callus started to be absorbed, and the cortical bone began to form in groups B, C, and D. While the cortical bone was hard to see in group A, in group B, the autogenous bone cannot be seen due to being absorbed. At 12 weeks after surgery, group B exhibited significant bone formation and bony union. In terms of bone formation and union, group C was close to group B and excellent to group D. The defect in group A was still evident, which indicated that the critical bone defect can not be repaired by itself. The radiographic scoring of the X-ray was measured with the result listed in Table 1. The assessment of repair defect demonstrated a statistically significant improved bringing of the defect with C compared to D. Groups C and D had higher scores than group A (*P* < 0.05), but lower than group B (*P* < 0.05).

**Table 1:** Lane-Sandhu radiographic scoring

Group	4 weeks	8 weeks	12 weeks
Group A	1.63 ± 0.31	2.62 ± 0.85	3.38 ± 0.76
Group B	4.95 ± 0.27	8.16 ± 0.35	10.76 ± 0.53
Group C	4.36 ± 0.48^a^	7.53 ± 0.67^a^	9.16 ± 0.36^a^
Group D	4.12 ± 0.52^a,b^	7.36 ± 0.43^a,b^	8.56 ± 0.55^a,b^

Group A as the control group, group B as the autogenous bone group, group C as n-HA/PLGA, and group D as β-TCP/PLGA. In the time point of 4, 8, and 12 weeks after surgery, compared to group A and group B, ^a^*P* < 0.05. Compared to group C, ^b^*P* < 0.05

### Histological analysis

**Fig. 2:**
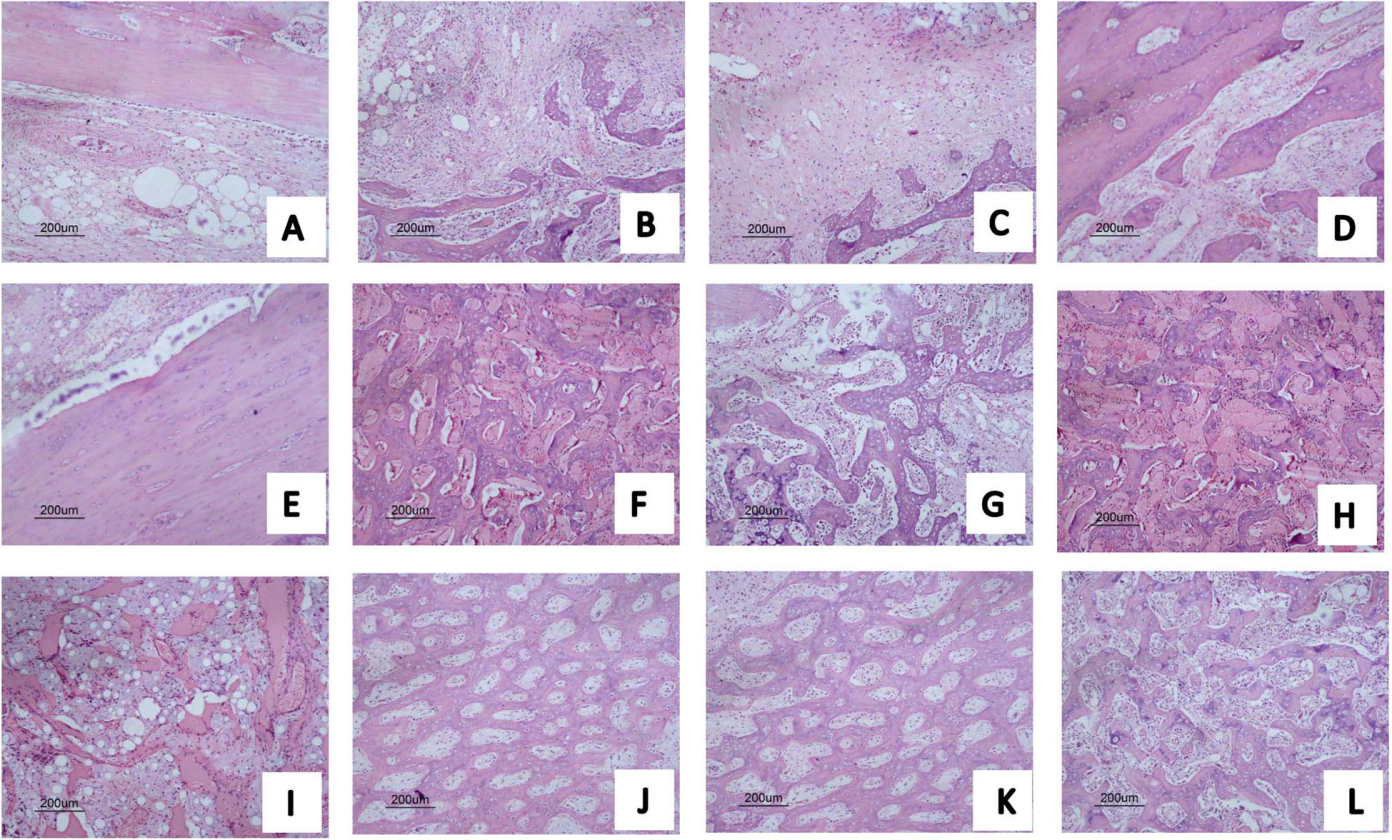
Hematoxylin and eosin staining for new bone tissue after operation 4 weeks (**a**), 8 weeks (**e**), and 12 weeks (**i**); B group after operation 4 weeks (**b**), 8 weeks (**f**), and 12 weeks (**j**); C group after operation 4 weeks (**c**), 8 weeks (**g**), and 12 weeks (**k**); and D group after operation 4 weeks (**d**), 8 weeks (**h**), and 12 weeks (**l**)

The bone formation in radius defect was evaluated by HE, which is displayed in Fig. 2. The histological analysis supported the X-ray result. Four weeks after the operation, there was no evident bone formation in group A; an amount of bone-like tissue formatted in defect with groups B, C, and D; and the scaffold in groups C and D degraded partially. After 8 weeks, the woven bone filled the defect areas in groups B, C, and D, and the scaffold in groups B, C, and D degraded completely. After 12 weeks, in group A, the defect without scaffold shows a loose connect tissue filled in the areas. The medullary canal in group B was recanalized. Defects in groups C and D show a larger number of woven bone formation. The new woven bone formation in defect areas in group C was larger than that in D.

### Immunohistochemistry analysis

**Fig. 3:**
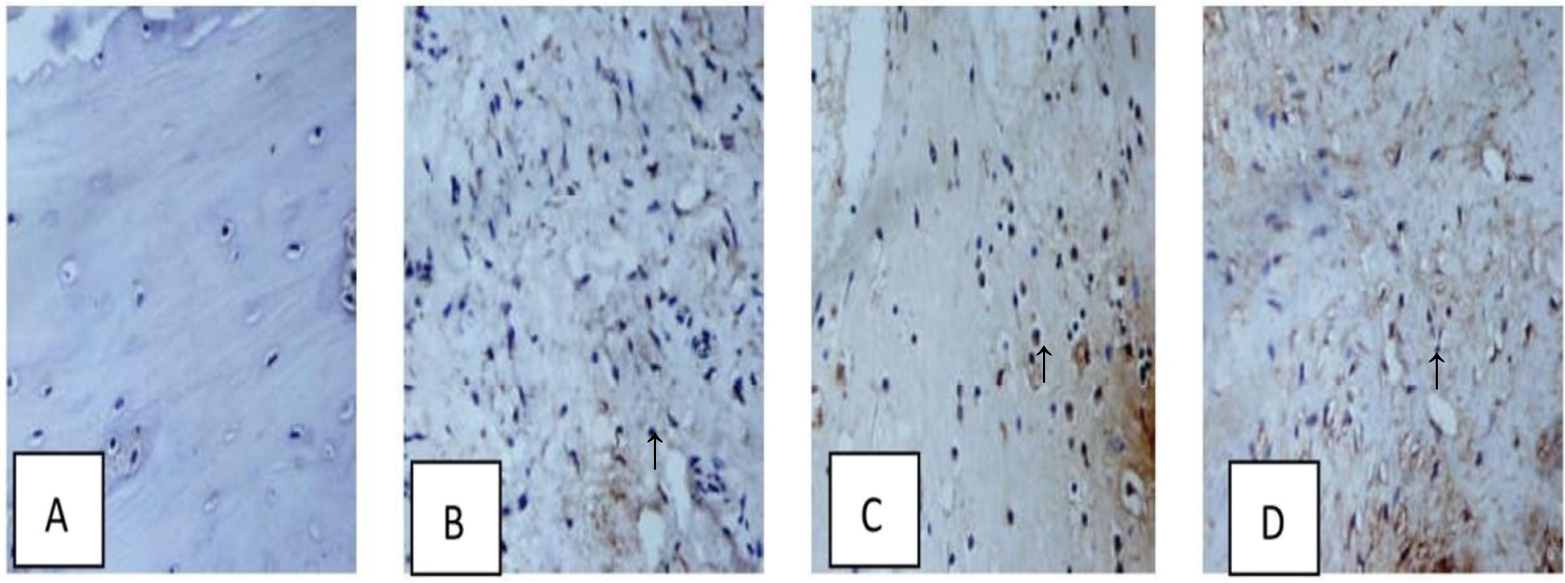
Immunohistochemical analysis for bone tissue after operation 4 weeks. There are positive tissue expression of OCN on the extracellular matrix (black arrow)

Immunohistochemical analysis was used to detect the expression of osteocalcin (OCN) within the positively stained area (brown color), during the early phase of bone repairing at 4 weeks. OCN protein expression was markedly upregulated in the defect area at 4 weeks. The expression of OCN is seen in all groups as shown in Fig. 3.

### Biomechanical results

The results of biomechanical testing are displayed in Table 2. The n-HA/PLGA and β-TCP/PLGA scaffolds enhanced the mechanical properties of the restored new bones as evidenced by a higher ultimate loading compared to group A. However, compared to group B, the biomechanics of the repaired radius by nHA/PLGA and β-TCP/PLGA were not sufficiently strong.

**Table 2:** Biomechanical performance of 4 groups 12 weeks after surgery

3-point bending test	Group A	Group B	Group C	Group D
Ultimate strength (N)	51 ± 1.6	121 ± 4.2	112 ± 5.5^a^	102 ± 2.3^a,b^

Group A as the control group, group B as the autogenous bone group, group C as the n-HA/PLGA group, and group D as the β-TCP/PLGA group. Compared to groups A and B, ^a^*P* < 0.05. Compared to C, ^b^*P* < 0.05

## Discussion

The repairment of bone defects using bone tissue engineering in an animal model has been proved to be effective [14]. In practice, the bone defect may suffer from seawater immersing in naval operation. It is vital to address tissue engineering and HBO in the field of seawater-immersed bone defects. Our study illuminates the effectiveness of repairing bone defect with n-HA/PLGA/BMSCs or β-TCP/PLGA/BMSCs combined with HBO.

Stem cells play a vital part in tissue engineering. In our study, allogenic bone marrow MSCs were used due to the following reasons: (1) autogenic MSCs were not recommended, because surgery on the iliac bone would lead to injury and pain. (2) Using allogenic MSCs did not lead to immunological rejection owing to the lack of expression of HLA class II antigens on MSCs [15]. (3) In the effectiveness of treating bone defect, there were similarities between autogenic MSCs and allogenic MSCs. Kang et al. [16] compare autogenic MSC with allogenic MSC in terms of bone regeneration in radius defect of the rabbits. Radiological, micro-CT, and histological analysis demonstrated no evidence of immune reaction in the allogenic MSC group. Meanwhile, allogenic MSCs possess a similar capacity for repairing defects compared to autologous MSCs.

The reasons for HBO improved bone healing in the study were enhancing vascularization, upregulating expression of osteogenic markers, and downregulating expression of pro-inflammatory cytokines [17].

Ideal scaffolds of tissue engineering have the following characteristics: good biocompatibility and appropriate degradation. PLGA approved by FDA in certain clinical applications [3] has been widely used in the treatment of bone defects. While PLGA has some disadvantages, limited abilities of osteoconduction and osteoinduction hinder the use in bone tissue engineering. However, the biggest disadvantage of PLGA is its acidic degradation products, which have lower pH values and easily cause inflammatory reactions in the implantation area. A lower pH value in the surrounding damages the proliferation of the cells, and the inflammatory reaction can trigger the release of cytokines by the host, damaging bone formation [18, 19]. The PLGA is an acidic degradation product, and n-HA and β-TCP are alkaline degradation products [20]. n-HA and β-TCP can mediate the pH produced by the acidic degradation products of PLGA. Therefore, they can avoid aseptic inflammation, which can provide a suitable microenvironment for new bone formation. He et al. [19] provide that nHA can neutralize the pH value due to the PLGA degradation. n-HA/PLGA revealed more strengthening effects in adhesion and osteogenic differentiation of BMSCs compared to PLGA [3].

A critical-sized bone defect is defined as the length of defect more than 1.5 times the diameter of the bone [21]. The length of the radius defect was 15 mm for the rabbit bone defect created in our experiment. The length was according to the bone defect model created by Wu et al. [22]. In the experiment of Wu et al., the rabbits of the 15 mm-radius defect in the control group which was implanted without any material cannot repair spontaneously. In our experiment, sixty New Zealand white rabbits were created a seawater-immersed bone defect model in their radius. After different therapeutic measures for correspondent groups, the injured limbs were gradually recovered. In our study, after 4 weeks of surgery, bone defect areas in groups C and D have low-density cloudy callus. After 12 weeks, there was an excellent connection and integration with broken ends, which displayed the composite of n-HA/PLGA and β-TCP/PLGA has an excellent repairment capacity for the seawater-immersed bone defect. There was no achievement of the complete bone union in group A. The three-point bending test showed that the mechanical properties in the n-HA/PLGA and β-TCP/PLGA group were closed to the autograft group.

## Conclusion

In the experiment, the histological and radiological study shows the scaffolds were degraded gradually and new callus was formed gradually and the radiographic result shows that the scaffold/MSC incorporation with HBO successfully repaired the 15-mm bone defect by 12 weeks after surgery. Scaffolds of n-HA/PLGA/BMSCs and β-TCP/PLGA incorporation with HBO were effective to treat seawater-immersed bone defect, and then n-HA/PLGA was more excellent than β-TCP/PLGA.

## Supplementary Information

**Additional file 1.**

## Abbreviations

BMSCs: Bone marrow mesenchymal stem cells
HBO: Hyperbaric oxygen
β-TCP: Beta tricalcium phosphate
PLGA: Poly lactic-co-glycolic acid
